# Evaluation of Driver’s Reaction Time Measured in Driving Simulator

**DOI:** 10.3390/s22093542

**Published:** 2022-05-06

**Authors:** Kristián Čulík, Alica Kalašová, Vladimíra Štefancová

**Affiliations:** 1 Department of Road and Urban Transport, University of Zilina, Univerzitna 1, 01026 Zilina, Slovakia; alica.kalasova@fpedas.uniza.sk; 2 Department of Railway Transport, University of Zilina, Univerzitna 1, 01026 Zilina, Slovakia; vladimira.stefancova@fpedas.uniza.sk

**Keywords:** driver behavior monitoring, driving simulators, road safety, ground vehicle safety

## Abstract

This article evaluates the driver’s reaction times in a driving simulator environment. The research focused mainly on young drivers under the age of 26, who cause many accidents. Each participating driver provided basic information later used for mathematical-statistical analysis. The main advantage of driving simulators is limitless usage. It is possible to simulate situations that would be unacceptable in real road traffic. Therefore, this study is also able to examine drunk driving. The main goal of the article is to evaluate if gender, practice, or alcohol significantly affected the reaction time of 30 drivers. We also focused on drinking before driving for a smaller number of the drivers; ten of them performed driving under the influence of alcohol. For these mathematical-statistical purposes, we used a one-sample *t*-test, a paired-samples *t*-test, an independent-sample *t*-test, and a correlation analysis together with the assessment of its statistical significance.

## 1. Introduction

In general, more than 90% of traffic accidents are caused by human failure [1,2,3]. A pedestrian, cyclist, or another road user can make a mistake. The driver causes the highest number of accidents. Fatigue and stress also contribute to road accidents [4]. Many studies focus on the behavior of professional drivers and their accidents [5,6]. Alcohol-related road accidents are also serious [7]. They often include young drivers, who are reckless and prone to alcohol consumption. Scientific studies define ‘young drivers’ differently. Most often they include drivers between the ages of 18 and 26. However, the upper limit may vary in some studies. For example, the authors in [8] focused on a group of 18 to 20-year-old drivers, the authors of the Romanian study in [9] focused on 18 to 24-year-old drivers, and the authors of the Belgian study in [10] focused on 17 to 24-year-old drivers. On the contrary, Dénommée et al. [11] focuses on 16 to 24-year-old drivers. Several studies have focused on the combination of young drivers and alcohol. In Greece, for example, the legal age for driving under the influence of alcohol is 18. A study of 241 young Greek drivers (aged between 18 and 24) found that young drivers whose dominant lifestyle was alcohol consumption were at a higher risk of being involved in an accident. In addition to alcohol, lack of driving experience also contributes to higher accident rates for young drivers [12].

Another study [13] has showed that male individuals are more likely to drink than female. Additionally, the authors in [14] have pointed out that after alcohol consumption, young male drivers tend to engage in risky behavior and aggression. Other studies, for example [15], have pointed to the differences in the characteristics of male and female drivers. Male risk perception is lower, which means they are more careless drivers [16,17]. A study [18] examined drivers’ reactions under the influence of alcohol. The main aim of this research was to investigate the effects of alcohol consumption on stopping behavior at stop signs and at an intersection with traffic lights. It was a laboratory experiment that also used a driving simulator. The results showed a significant difference between the mean deceleration values for sober drivers and drivers under the influence.

Alcohol impairs the judgment ability of the driver. It causes delayed responses, such as an increased reaction time when encountering a stimulus on the road [19,20]. A study [21] has also shown interesting results; blood alcohol concentration levels of 0.03%, 0.05%, and 0.08% resulted in 36%, 53%, and 94% incremental changes in the reaction times of drivers encountering a pedestrian crossing.

Many studies have showed that blood alcohol concentration is related to accident risk [22,23]. Young drivers who drive under the influence of alcohol have a higher risk of accident involvement at all blood alcohol concentration levels [24,25]. Many authors have proved that sober drivers represent a lower accident risk [26,27].

It is possible to find many theoretical studies that deal with traffic accidents. They focus on traffic accidents correlated with the driver’s age or alcohol consumption. It is problematic to carry out research in real road traffic in this area. It is possible to use a driving simulator to cover this research gap. Young drivers in the age group of 18 to 26 are particularly problematic; hence, this demographic was chosen.

The research described in this article had several main objectives. The first task was to measure the accurate values of the drivers’ reaction times. The evaluation aimed to consider differences between the reaction times of:

1. drivers that do not expect obstacles on the road and drivers on the second attempt when they know the scenario,
2. male and female drivers,
3. sober drivers and drivers under the influence of alcohol.

The secondary objective was to describe and evaluate the best possibilities for enhancing the simulation validity. Simulator improvements can bring better results and eliminate simulator sickness.

## 2. Materials and Methods

One of the research aims was to measure the reaction times of drivers under the influence of alcohol. This measurement would not be possible in real road conditions. Therefore, a driving simulator was used. As it is a complex device, it is described in detail in the following section.

### 2.1. Driving Simulators

A driving simulator can be simply defined as a device that simulates the driving of a road vehicle in a virtual environment as realistically as possible [28,29,30].

An advanced driving simulator should reproduce all stimuli that the driver perceives when driving. The simulator software should ensure [31]:

- mathematical model of vehicle behavior,
- virtual reality—image and sound,
- scene control/event generator,
- platform movement control,
- driving record,
- tools for evaluating driver’s behavior.

The University Science Park at University of Žilina has a training driving simulator available for research purposes. The essential difference between the research driving simulator and the training driving simulator is in their purposes:

(a) Training driving simulators, usually used in driving schools, are devices used for training new drivers. The term ‘training driving simulator’ can be found in Methodical Instruction no. 22/2005 on technical requirements for training driving simulators [32] from 26 September 2005. This guideline sets out the basic requirements for training driving simulators. There is, for example, a requirement for a projection area of at least 180 × 130 mm^2^ and other conditions for sound, vehicle dynamics, and virtual environment. Researchers use training driving simulators occasionally, for example, in the study [33] or for evaluation of training effectiveness in driving schools [34].

(b) Research driving simulators have a wide range of use in research institutes, universities, and the automotive industry. Their main advantage is the ability to adapt to the current requirements of the experiment. It means that they must have an open system in which it is possible to change the virtual environment, vehicle, and its properties. They can be used, e.g., for driver fatigue research [35,36], crossing intersections [37], lane change behavior [38,39,40], driver error rate [41], driver glare [42], or human-vehicle interaction research in general [43]. Simulators also have great potential in autonomous vehicle research [44,45,46].

Driving simulators have various advantages and disadvantages depending on the individual versions and construction arrangements. The advantages of driving simulators include, for example [20]:

- Versatility and new developments at reduced cost. Simulators can be easily and economically configured to research many human factor issues.
- Experimental control and measurement. Driving simulators allow researchers to control experimental conditions and measure any parameters. For example, a study [47] measured steering wheel angles while changing lanes when the gap between vehicles in the target lane was constant or decreasing, as well as maneuvering times. These were subsequently projected into a graphic form.
- Safety. Driving simulators provide a safe environment for driver research.

Driving simulators also have several disadvantages—weaknesses that every researcher should consider as a limitation of the study:

- Validity. Simulators cannot duplicate the whole world due to its details and complexity. Therefore, this raises the question of to what extent the research on a simulator is credible. Some authors have described this issue in the article [48], which compares 44 studies. Another comprehensive study is in [20]. The virtual environment can be very different or very similar to real conditions. A study [39] has evaluated the similarity between real driving and driving in a simulator. Interestingly, it showed similar results between simulation and reality (similar measured speeds in turning and connecting lanes).
- Costs. Driving simulators have relatively high acquisition costs, but very low operating costs.
- Simulator sickness [49]. Usually, driving simulators with a motion system or poor graphic quality cause nausea. These impacts on the human body are so-called Simulator Adaption Syndrome (SAS). The authors of [50,51] have written that the source of SAS was the difference between the performances of the driving simulator and the real vehicle. Many studies, for example [52,53], have compared the negative effects of static and motion simulators. According to them, the most common symptoms are nausea (feeling sick), dizziness, vomiting, eye pain, fatigue, and anxiety. Interestingly, they are less common in dynamic (moving) simulators.

In our research, the SNA–211 REN training driving simulator was used for experimental driving [54,55]. The participants in this study had not yet had experience with this driving simulator. Therefore, they had to be trained before their performance could be measured. Drivers had about 10 min to familiarize themselves with the environment and the simulator controls. Each driver could try starting, braking, turning, and shifting between gears.

### 2.2. Other Usedequipment

**Figure 1:**
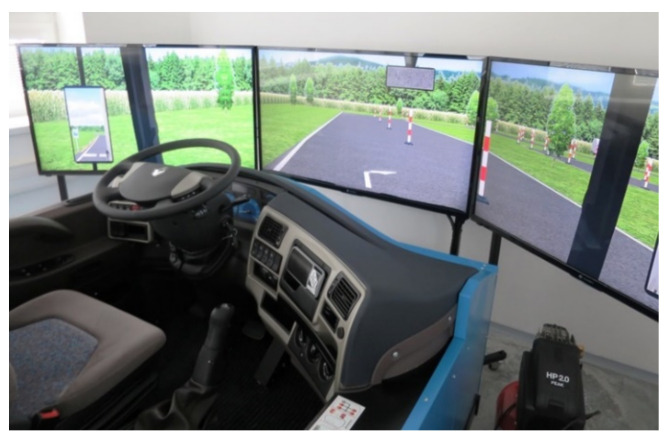
Training driving simulator in SNA–211 REN. Source: Processed by authors.

The central element of the research is the driving simulator (Figure 1). It is a replica of a truck cabin equipped with a gear stick with a small button. It can switch gears between the lower row (1st to 4th gears) and the upper row (5th to 8th gears).

There were three people in the laboratory during the individual measurements: the supervisor of the driving simulator, the person responsible for data recording, and the tested driver. The other participants involved in the research task were in another room during the measurements. It was necessary to ensure that they had not seen the obstacles and virtual environment before their ride.

**Figure 2:**
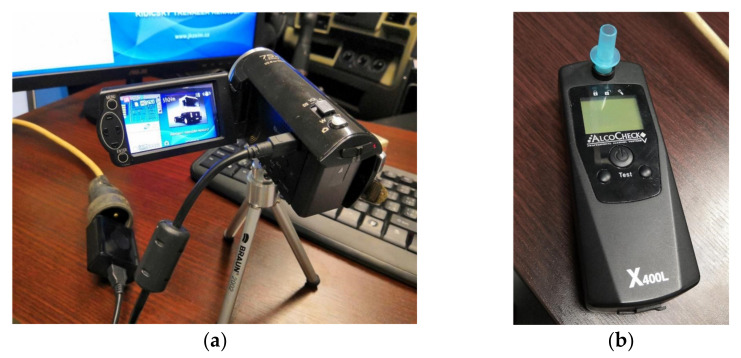
Other equipment: (**a**) External video camera for recording; (**b**) AlcoCheck X400L. Source: Processed by authors.

The drivers’ reactions encompassed the moment when an unexpected situation occurred until the moment when they activated the brake pedal. The computer program Corel VideoStudio recorded the environment and braking. It was installed on the driving simulator computer. The recording was also made using an external camera (Figure 2a). An AlcoCheck X400L (Figure 2b) was also used to test alcohol in the drivers’ breath. The obtained values were not used in statistical evaluation.

### 2.3. Measurement Methodology

In our research, we evaluated the reaction times of 30 drivers. Half of them were male drivers and half of them were female drivers. The average ages of the male and female drivers were 22.27 years (SD = 1.58) and 22.33 years (SD = 1.84), respectively. The average mileages of the male and female drivers were 45,200 km (SD = 34,526) and 16,700 km (SD = 15,780), respectively. 

Measurement was divided into five parts, which are described in detail in the following lines.

#### 2.3.1. First Part of Experiment: Unexpected Obstacle

For the first measurement, the drivers were focused, but did not expect an obstacle. The obstacle in the virtual environment was an animal running from behind a tree across the road. The reaction time of the driver was recorded as the time interval between the obstacle animation trigger and the moment of brake activation. We did not evaluate the success of the obstacle stop in this article.

#### 2.3.2. Second Part of Experiment: Expected Obstacle

The second measurement took place at the same time as the first. The scenario for measuring reaction times continued, but the drivers were already expecting another obstacle. Therefore, the reaction time should be even shorter than in the case of the first sudden obstacle. For this measurement, the drivers were more careful. They peripherally checked the edge of the road.

#### 2.3.3. Third Part of Experiment: Impressions from the Simulation

Given the need to increase the validity of the simulation in the future, after completing these two measurements, all test drivers completed a questionnaire. It aimed to record the perceived quality of the simulation. For most drivers, this was the final part of the measurement. A minority also took part in the fourth and fifth parts of the experiment: driving after drinking alcohol.

#### 2.3.4. Fourth Part of Experiment: Drunk Driving 1

For technical reasons, not all drivers performed further tests. Therefore, only 10 drivers were chosen for the last part of the measurement. All drivers involved in the fourth and fifth parts of the experiment agreed to drink alcohol. Without accounting for the differences in weight and other factors, each driver consumed 200 mL of 35% alcohol. Each driver had 10 min for this consumption. After a subsequent 10-min break, his or her ride began in a virtual environment. The drivers’ responses to a sudden obstacle was measured again. The scene was the same as in previous measurements.

#### 2.3.5. Fifth Part of Experiment: Drunk Driving 2

This measurement was taken after a long break from the time of first alcohol consumption (30 min) to increase its influence on the drivers’ behavior and attention. The drivers went through the same scene. Here we expected a more significant deterioration of their reactions and a higher level of alcohol in their breath.

Before the measurement process, it was necessary to ensure:

1. Drivers signed the Informed Consent agreement before the experiment.
2. Familiarization with the course of research.
3. Drivers who had to undergo drunk driving had to consume no alcohol before driving, be in approximately the same sleep mode (students from the same study group who get up at the same time). The had to consume the same food (lunch together with the same menu).

The procedure of the measurement itself was as follows:

1. Familiarization of the driver with the driving simulator (10 min).
2. Start of the scenario for measuring reactions no.1 (15 min).

- Measurement of the time interval between the trigger start time and the activation of the brake pedal.

3. Start of the reaction measurement taken during scenario no. 2 (5 min).

- Measurement of the time interval between the trigger start time and the activation of the brake pedal.

4. Completion of the simulation validity questionnaire (10 min).

- The questionnaire was in paper form.

5. Alcohol consumption.
6. 10 min break.
7. Start of the scenario to measure reactions no. 3 (5 min).

- Measurement of the time interval between the trigger start time and the activation of the brake pedal.

8. Break 15 min.
9. Start of the scenario to measure reactions no. 4 (5 min).

- Measurement of the time interval between the trigger start time and the activation of the brake pedal.

10. End of measurement.

### 2.4. Evaluation Methods

Using software, such as SPSS or the Data Analysis add-in in MS Excel, it is possible to evaluate reaction times in a modern and simple way. However, in this article, the authors used the methods described below, calculated in the traditional way, to demonstrate the possibilities of statistical evaluation. The following methods will therefore be used to evaluate reaction times [56]:

- One-sample *t*-test. We use one-sample *t*-test in experimental situations where we know the mean value µ_0_ of the basic set. We can then consider this as a constant. In this experiment, we verify the hypothesis that the experimental sample comes from a population that has the same mean as this known constant. We test the null hypothesis: H_0_: *µ*_0_ = *const.* We start the test from the data of the monitored sample, which we assume comes from a population with certain parameters *µ* and s^2^ and further from the known mean value of the base set m, which is equal to a certain (known) constant.
- Two-sample *t*-test. This test evaluates experiments where we do not know the mean of the base set and compares only two sets of sample data. These data can be represented by either two measurements performed repeatedly on one group of individuals (paired experiment) or by two independent groups of measurements (non-paired experiment). In the case of a two-sample *t*-test, we test the null hypothesis: *H_0_: µ*_1_ *= µ*_2_. A two-sample *t*-test can be:

- Independent-sample *t*-test, which compares the data formed by two independent selections, i.e., that they come from two different groups of individuals. Typically, this is a comparison of the values of the experimental group (where the experimental intervention was applied) and the control group (where the experimental intervention was not performed).
- Dependent-sample *t*-test, which compares the data that make up “paired variation series,” i.e., where they come from those subjects that were subjected to two measurements.

- Correlation analysis. This simple correlation analysis deals with the evaluation of the dependence of two random variables and emphasizes the intensity of the relationship rather than the examination of variables in a cause-effect relationship (regression) [57].
- Correlation coefficient significance test. A common task in mathematical statistics is to find out whether the random variables X and Y are correlated or not. The value of the correlation coefficient depends on the elements in the random selection. If the value of the correlation coefficient is close to zero, we want to verify whether it is only random (caused by random selection) or whether it is really a linear independence. The linear independence test is used for verification. We express the hypothesis H_0_: ρ = 0 against the alternative hypothesis H_1_: ρ ≠ 0 to find out whether the random variables X, Y are correlated or not [58].

### 2.5. Reaction Time Values and Hypothesis

In this article, we use a *t*-test to test the hypotheses described below. First, we verify that the mean value of the reaction times in the first measurement is equal to 0.8 s, which is located in the middle of the table below, signifying the concentrated drivers who do not expect the stimulus (Table 1). We assume that the mean value is less than the table data, and we verify this hypothesis at the significance level α = 5%.

We further verify the hypothesis that the mean reaction time for male and female drivers does not differ at the 5% significance level. We verify this hypothesis using an independent-sample *t*-test, performed for the first and second measurements.

Third, we use the Dependent-Sample *t*-test to verify the research hypothesis that the mean reaction time before alcohol consumption is less than the mean value of the reaction time after alcohol consumption. We verify this hypothesis on 20 values measured during the first and second phases, and 20 values measured under the influence of alcohol in the fourth and fifth phases of the research.

In the last part, we calculate the correlation coefficient between the mileages and the driver’s reaction time. In addition, we verify the statistical significance of the correlation coefficient. We decide whether the detected dependence (regardless of the value of the correlation coefficient) is statistically significant or random. We perform all the above tests at the significance level α = 5%.

## 3. Results

In total, we were able to measure the reaction times of 30 drivers at two points in time. Drivers also completed a questionnaire on simulation impressions. All data measured during these experiments are presented in the individual tests in the following sections. 

### 3.1. One-Sample t-Test

**Figure 3:**
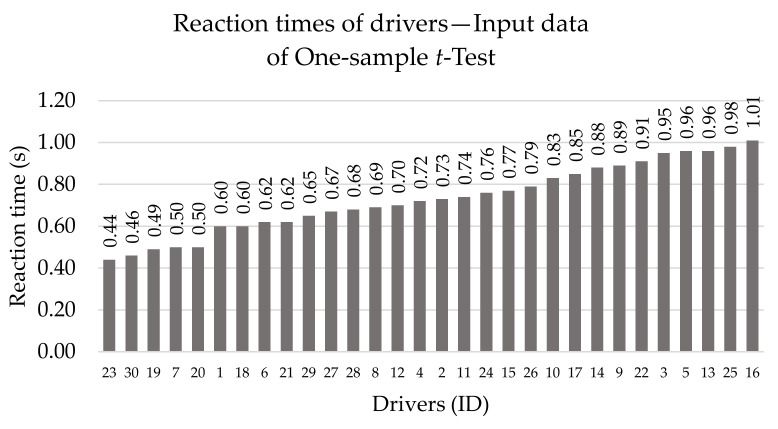
Input data of one-sample *t*-test. Source: Processed by authors.

We have organized the individual tests into different sections, so that, in addition to the results themselves, we can also point out our methods for statistical testing. We performed the one-sample *t*-test with the data visualized in the Figure 3.

The procedure for testing with the one-sample *t*-test is as follows:

1. Determination of hypotheses:
  **H_0A_**  : *The mean value of the reaction times of concentrated drivers is 0.80 s: µ = 0.80 s.*
  **H_1A_**  : *The mean value of the reaction times of concentrated drivers is less than 0.80 s: µ < 0.80 s.*
2. Calculation of test criterion (1), in which $\bar{x}$ is the arithmetic mean of all measured values of reaction times (0.732) and $\mu_{0}$ is chosen as 0.80 s. In the equation, n is the number of all measurements (30) and S is standard deviation (0.166).
  (1) $$t=\frac{\bar{x}-\mu_{0}}{S}\cdot \sqrt{n}$$
  After substituting, we find that the test criterion has a value −2.252.
3. The critical field is presented in the formula (2), where α is the level of significance, in our case 0.05. Subsequently, we looked in the quantile tables of the Student’s distribution for the value $t_{0.95}\left(29\right)$, which is 1.699.
  (2) $$W_{\alpha }=\left\{t\leq -t_{1-\alpha }\left(n-1\right)\right\}$$
  Subsequently, we can complete the formula as follows (3):
  (3) $$W_{\alpha }=\left\{-2.252\leq -1.699\right\}$$
  From this, we can conclude that the critical field is fulfilled and thus, we reject the original hypothesis H_0A_ and accept the alternative hypothesis H_1A_.
4. The answer in this case is: the mean value of the reaction times of the concentrated drivers is less than 0.80 s at a significance level of 5%.

### 3.2. Independent-Sample t-Test

**Figure 4:**
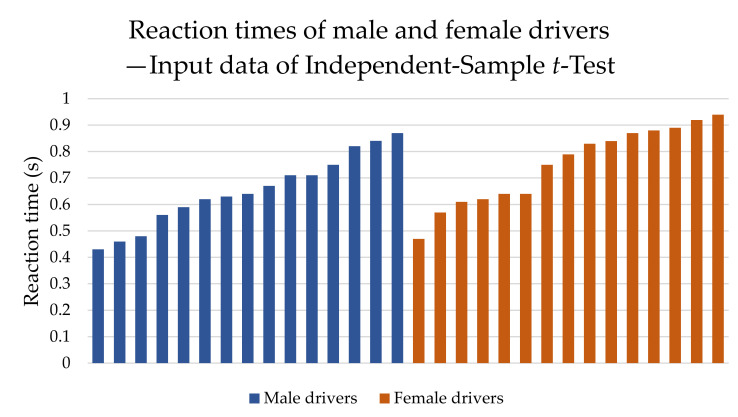
Input data of independent-sample *t*-test. Source: Processed by authors.

An independent-sample *t*-test was the second test that we used. This test for independent selections is a commonly used method to evaluate the difference in the averages of the two groups. The test is used for small samples on the assumption that both groups have a normal distribution and the variances of these groups do not differ significantly. The input data are visualized in Figure 4.

The procedure for testing with the independent-sample *t*-test is as follows:

1. Determination of hypotheses:
  **H_0B_**  : *The mean reaction time of male and female drivers is the same*: µ_M_ = µ_W_.
  **H_1B_**  : *The mean value of the reaction time of male and female drivers is not the same:* µ_M_ ≠ µ_W_.
2. Calculation of test criterion (4), in which $\bar{x_{1}}$ and $\bar{x_{2}}$ are the arithmetic means of all measured values of reaction times of males and females, respectively. The number of measurements is denoted as $n_{1}$ and $n_{2}$. In the case of this test, the measurement values may also be different, as they are not paired. $S_{1}$ and $S_{1}$ are the standard deviations, which are $S_{1}=0.147$ and $S_{2}=0.136$.
  (4) $$u=\frac{\bar{x_{1}}-\bar{x_{2}}}{\sqrt{\frac{S_{1}^{2}}{n_{1}}+\frac{S_{2}^{2}}{n_{2}}}}$$
  After substituting, we find that the test criterion has a value +1.910.
3. The critical field in this case is given by (5), where α is the level of significance (0.05). Subsequently, we looked in the quantile tables of the normal distribution N (0,1) for the value $u_{0.975}$, which is 1.960.
  (5) $$W_{\alpha }=\left\{\left|u\right|\geq u_{1-\frac{\alpha }{2}}\right\}$$
  Subsequently, we can complete the formula of critical field as follows (6):
  (6) $$W_{\alpha }=\left\{1.910<1.960\right\}$$
  From this, we can conclude that the critical field is not met and therefore we accept the original hypothesis H_0B_.
4. The answer in this case is: the mean reaction time of male and female drivers is the same at a significance level of 5%. However, as it can be seen, the test criterion is very close to the critical range.

### 3.3. Paired-Samples t-Test

**Figure 5:**
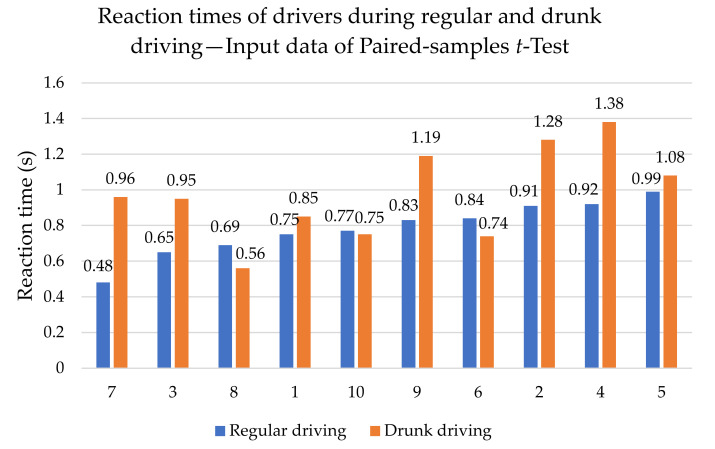
Input data of paired-samples *t*-test. Source: Processed by authors.

The paired-samples *t*-test was the third one we used. This test compares the values of a variable for the same respondent in two different experimental conditions. In our case, we use this test to compare the reaction time before and after drinking alcohol. Analyzed reaction times are visualized in Figure 5.

The procedure for testing with the paired-samples *t*-test is as follows:

1 Determination of hypotheses:
  **H_0C_**  : *The mean value of the reaction times of the drivers in a sober state and under the influence of alcohol is the same: µ_S_ = µ_D_.*
  **H_1C_**  : *The mean value of the reaction times of drivers in a sober state and under the influence of alcohol is not the same: µ_S_ ≠ µ_D_.*
2. Calculation of test criterion (7), in which $\bar{D}$ is the arithmetic mean of all mutual deviations (differences) between two experiments. The number of measurements is denoted as *n*. It is also necessary to calculate the standard deviation $S_{D}$ from all values of the mentioned differences for the calculation of the test criterion.
  (7) $$T=\frac{\sqrt{n}\cdot \bar{D}}{S_{D}}$$
  After substituting, we find that the test criterion has a value −2.618.
3. In this case, the critical field is given by (8), where α is the level of significance (0.05). Subsequently, we looked in the quantile tables of the Student’s distribution for the value $t_{0.975}\left(9\right)$, which is 2.262.
  (8) $$W_{\alpha }=\left\{\left|t\right|\geq t_{1-\frac{\alpha }{2}}\left(n-1\right)\right\}$$
  Subsequently, we can check (9) the fulfillment or non-fulfillment of the critical field:
  (9) $$W_{\alpha }=\left\{2.618>2.262\right\}$$
  From (9) we conclude that the critical field is fulfilled and thus we reject the original hypothesis H_0C_ and accept the alternative hypothesis H_1C_.
4. The answer in this case is: at a significance level of 5%, it was shown that the mean values of the reaction times of drivers in a sober state and under the influence of alcohol are not the same.

### 3.4. Correlation Coefficient Test

The last test, a Correlation Coefficient Test, assesses the statistical significance of the correlation between two variables. It should be noted that relatively low values of correlation coefficients can be expected in traffic psychological research. According to [38], the interpretation of the correlation coefficient depends on the context. In field of physics, a correlation coefficient of 0.8 is very low; on the contrary, in the social sciences, it is a very high value. In 1988, Cohen [60] established the exact tool for the interpretation of correlation coefficients in psychological research:

- A correlation in the absolute value below 0.1 is trivial,
- A correlation in the range of 0.1 to 0.3 is small,
- In the interval of 0.3 to 0.5, the correlation is medium,
- At values above 0.5, the correlation is high,
- A correlation of 0.7 to 0.9 is very high,
- A correlation in the range from 0.9 to 1.0 is almost perfect.

The correlation coefficient (10) measures the two-tailed linear dependence of two variables and takes values from the interval 〈−1;1〉. The following implications apply for the correlation coefficient:

r_xy_ = 0 ⇔ variables X and Y are not linearly dependent,

r_xy_ > 0 ⇔ there is a direct linear relationship between the variables X and Y,

r_xy_ < 0 ⇔ there is an indirect linear relationship between the variables X and Y.

The sign of the correlation coefficient determines the direction of the dependence. The absolute value of the correlation coefficient reveals the strength of the linear association between the two variables. The closer the absolute value is to 1, the stronger the dependence.

(10) $$r_{xy}=\frac{n\sum xy-\sum x\sum y}{\sqrt{\left[n\sum x^{2}-{\left(\sum x\right)}^{2}\right]\cdot \left[n\sum y^{2}-{\left(\sum y\right)}^{2}\right]}}$$

**Figure 6:**
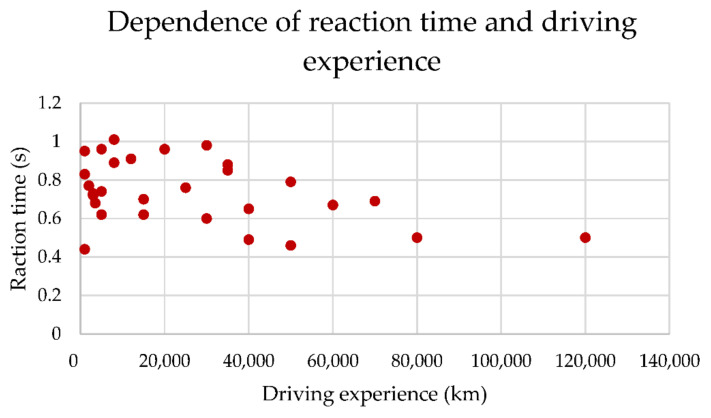
Input data of correlation analysis. Source: Processed by authors.

In this case, we will calculate the correlation coefficient between the number of traveled kilometers x. (driving experience) and the reaction time of drivers y on the first attempt. The correlation coefficient can be very easily calculated in MS Excel using the CORREL function. In our study, the correlation coefficient was −0.430, which can be considered a non-proportional medium dependence. The described dependence is visualized in the Figure 6.

The procedure for testing the correlation coefficient is as follows:

1. Determination of hypotheses:
  **H_0D_**  : *There is no statistically significant linear relationship between the variables y and x*.
  **H_1D_**  : *There is a statistically significant linear relationship between the variables y and x*.
2. Calculation of test criterion (11), in which r is the correlation coefficient calculated above and n is the number of all data pairs (30).
  (11) $$T=r\cdot \sqrt{\frac{n-2}{1-r^{2}}}$$
  After substituting, we find that the test criterion has a value −2.522.
3. The Critical Field is given by (12), where α is the level of significance (0.05). Subsequently, we looked in the quantile tables of the Student’s distribution for the value value $t_{0.975}\left(28\right)$, which is 1.699.
  (12) $$W_{\alpha }=\left\{\left|t\right|\geq t_{1-\frac{\alpha }{2}}\left(n-2\right)\right\}$$
  Subsequently, we can add to the formula itself as follows (13):
  (13) $$W_{\alpha }=\left\{2.522>2.048\right\}$$
  From this, we can conclude that the critical field is fulfilled and thus, we reject the original hypothesis H_0D_ and accept the alternative hypothesis H_1D_.
4. The answer is: At a significance level of 5%, it was shown that there is a statistically significant linear relationship between the variables y and x.

**Figure 7:**
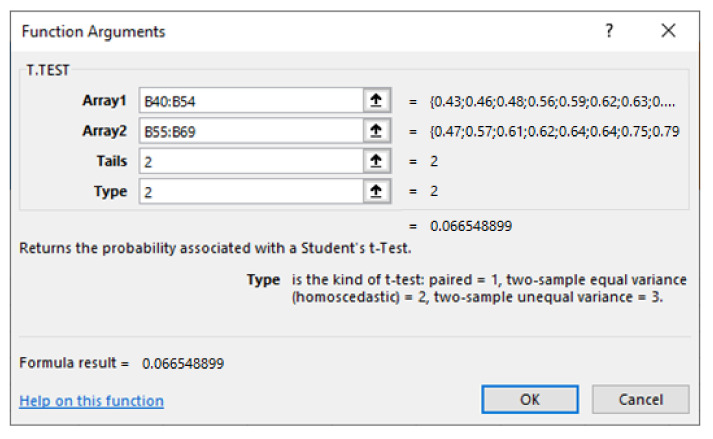
Independent sample *t*-test with T.TEST function in MS Excel. Source: Microsoft Excel.

We have calculated all these tests in statistical software. The most commonly used program is SPSS, but it is also possible to perform these tests in MS Excel with the T.TEST function. The following figure (Figure 7) shows an example of the Independent Sample *t*-test setting in MS Excel (Array1—reaction times of male drivers, Array2—reaction times of female drivers, Tails 2—two-tailed test, type 2—the variances do not differ).

## 4. Discussion

**Figure 8:**
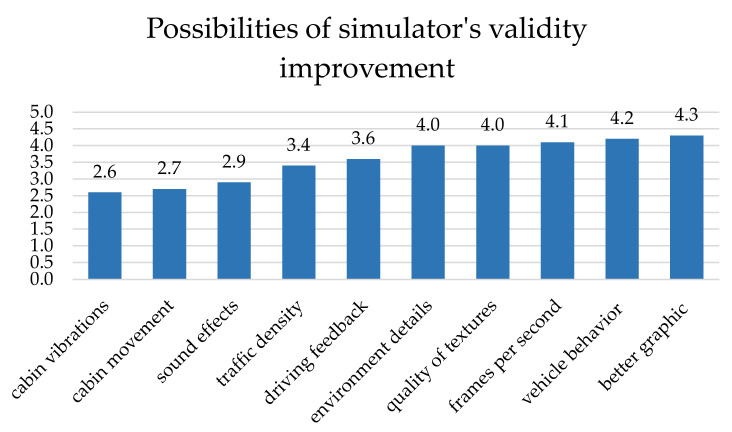
Possibilities of improving driving simulator validity. Source: Processed by authors.

This article describes the measurement of drivers’ reaction times in the driving simulator. Its essential goal was to point out the possibilities of statistical evaluation of the measured values. Due to the equipment available for this research, the driving simulator can be considered a limitation of the study. As can be seen from the graph in the following figure (Figure 8), the drivers also took part in a survey of the perception of virtual reality in the third phase of testing. In the survey, they evaluated what could make the ride more realistic (1 = the least significant factor, 5 = the most important improvement). It is clear from the picture that the graphics and behavior of the vehicle most significantly contribute to the perception of reality. Other related factors are the number of frames per second, the quality of the textures, and the traffic in the virtual environment [61]. Drivers also mentioned vehicle behavior and steering response. Another study [62] deals with the possibilities of eliminating such defects in driving simulators.

Figure 8 shows that drivers perceive the vibrations and movements of the simulator’s cab as most insignificant. Financially, these two requirements would be the most economically demanding. The authors of [63,64,65] write that cabin movements can increase the validity of the simulation. However, at the same time, they can cause negative feelings from driving. In these studies, the authors compared three simulator options: a fixed base platform with poor visibility, a fixed base platform with good visibility, and a motion base platform with good visibility. It is clear from the studies that most health problems, such as nausea, oculomotor, and disorientation, occur when using the motion platform.

In terms of the experiment itself, in this study, we were able to evaluate the reaction times of 30 drivers, which is not a sufficiently representative sample. In Slovakia, there are 244,663 registered drivers in the 17–24 age group. According to a sample size calculator, with a population of 244,663, a confidence level of 95%, and a confidence interval of 5, we would need a sample size of 384 people. However, this was not possible in our case. For this reason, we verified the statistical significance by testing hypotheses.

Another goal of the paper was to provide educational value. This article shows how it is possible to statistically evaluate data. Therefore, students at universities can use the described methods for their theses. For this reason, all four tests are performed not by software (through the so-called *p*-values), but by traditional calculations.

The research described in this article has several limitations. First, it would be necessary to ensure approximately the same physiological training for drivers, especially those who also took part in driving under the influence of alcohol. The drivers who also took part in the second part of the research were students from one study group, thus ensuring they had a similar duration of regular sleep and a similar diet. However, we could not completely monitor all consumed meals.

From the results and the literature, it is obvious that alcohol has a significant effect on drivers’ reaction times and overall behavior. We did not record detailed physiological data on the individual drivers during our research. A detailed comparison of measured values would be problematic. Characteristics, such as gender, physical condition, and especially the weight of a person, significantly affect the coping of the human body with the same dose of alcohol consumed. Therefore, for further research in this area, we recommend the physiological preparation of drivers (sleep, drug exclusion, etc.) and a thorough investigation and recording of the relevant characteristics of the individual persons involved. Other than age and gender, we did not evaluate any other drivers’ attributes. It is a limitation of the study.

Another limitation of the study is that we used the training driving simulator, which is a replica of the truck Renault Midlum. For measurements, a passenger car would be more appropriate. On the other hand, there was no difference in driving, because all drivers used only four high gears. There was no problem driving with only four gears and an unloaded vehicle.

This research provided several results. In the first test, the tabular value of the reaction time of the concentrated driver, who did not expect a stimulus, was not completely confirmed. The reaction times were shorter, probably due to the lack of distractors during the drive.

Psychological research in this paper did not consider the drivers’ hand preferences. According to recent studies, this area seems to be very interesting, and we can address it in our next scientific endeavor. Due to professional contributions, left-handed people used to be routinely excluded from studies. Hand preference is problematic, but at the same time it is a very useful variable that deserves its place in the deeper examination of human behavior.

Statistical testing also confirmed that the reaction time of male and female drivers is approximately the same. However, in this case, it is possible that similar reaction times occurred due to the presence of similar people in the selected sample. All the young drivers were students or graduates of a technical university. It means that this could have an impact on the results.

With the paired-samples *t*-test, we tested the hypothesis of prolonging the reaction times of drivers under the influence of alcohol. The reaction times were indeed even shorter in some attempts. However, in general, at a significance level of 0.05, it can be stated that the times are different and, of course, shorter in a sober state.

The last point of the evaluation was the correlation analysis. Calculating the correlation coefficient without assessing its statistical significance can bring misleading results. In our case, the correlation coefficient between driving experience and reaction time was a mean inverse value of −0.430. In the field of physics, this would be a low value. However, in traffic psychology, this represents a medium dependence. The Correlation Coefficient Test proved a statistically significant linear relationship between the above variables at the 5% significance level.

## 5. Conclusions

This article focused on the drivers’ reaction time measurements while driving in the simulator. For the measurement process, we formulated the following recommendations:

- Measurement accuracy is a critical factor because the reaction time is a short time interval.
- It is necessary to avoid time delays caused by slow response time. These delays arose from hardware and should be avoided.
- It is also crucial to ensure that individual respondents do not provide information about the process of the experiment.
- Drivers should be in approximately the same psycho-physiological condition.
- From the research results, we can formulate the following recommendations:
- The consumption of alcohol before driving prolongs reaction time and thus increases the risk of an accident. Therefore, it is necessary to protect young drivers through prevention campaigns.
- Drivers with higher mileage have a better reaction time, but only in some cases (correlation coefficient 0.430).
- Concentration during driving significantly shortens the reaction time. Therefore, the main recommendation of the study is to maintain attention while driving.

From this article, the danger of drunk driving is evident. However, it is also clear from many other studies [65,66,67,68]. In terms of statistics, we have pointed out that the basic characteristics (median, mode, arithmetic mean, or standard deviation) are insufficient in similar research. It is necessary to assess statistical significance.

## Figures and Tables

**Table 1:** Drivers’ reaction times for different conditions. Source: [59].

Reaction Time [s]	Driver
0.6–0.7	driver is attentive, focused, awaiting stimulus and ready to brake
0.7–0.9	driver is attentive, but does not expect a stimulus
1.0–1.2	driver has focused his or her attention on other activities related to driving (driving, preventing, sidewalk observation)
1.4–1.8	driver is inattentive (having fun with the passenger, etc.)
1.6–2.4	driver is indisposed (alcohol, illness, fatigue, etc.)

## Data Availability

All used data is available on request from the author.
